# Graphene Oxide Bionanocomposite Coatings with High Oxygen Barrier Properties

**DOI:** 10.3390/nano6120244

**Published:** 2016-12-21

**Authors:** Ilke Uysal Unalan, Derya Boyacı, Masoud Ghaani, Silvia Trabattoni, Stefano Farris

**Affiliations:** 1 DeFENS, Department of Food, Environmental and Nutritional Sciences—Packaging Division, University of Milan, via Celoria 2-20133 Milan, Italy; iuysalunalan@gmail.com or uysaluna@msu.edu (I.U.U.); boyaci.derya@gmail.com (D.B.); masoud.ghaani@unimi.it (M.G.); 2 Department of Food Engineering, Faculty of Engineering, İzmir University of Economics, İzmir 35330, Turkey; 3 School of Packaging, Michigan State University, East Lansing, MI 48824, USA; 4 Department of Food Engineering, Izmir Institute of Technology, İzmir 35430, Turkey; 5 Department of Materials Science, University of Milano Bicocca, via Cozzi 55-20125 Milan, Italy; silvia.trabattoni@mater.unimib.it; 6 INSTM, National Consortium of Materials Science and Technology, Local Unit University of Milan, via Celoria 2-20133 Milan, Italy

**Keywords:** graphene oxide, haze, modelling, oxygen transmission rate, pullulan, relative humidity

## Abstract

In this work, we present the development of bionanocomposite coatings on poly(ethylene terephthalate) (PET) with outstanding oxygen barrier properties. Pullulan and graphene oxide (GO) were used as main polymer phase and nanobuilding block (NBB), respectively. The oxygen barrier performance was investigated at different filler volume fractions (ϕ) and as a function of different relative humidity (RH) values. Noticeably, the impermeable nature of GO was reflected under dry conditions, in which an oxygen transmission rate (*OTR*, mL·m^−2^·24 h^−1^) value below the detection limit of the instrument (0.01 mL·m^−2^·24 h^−1^) was recorded, even for ϕ as low as 0.0004. A dramatic increase of the *OTR* values occurred in humid conditions, such that the barrier performance was totally lost at 90% RH (the *OTR* of coated PET films was equal to the *OTR* of bare PET films). Modelling of the experimental *OTR* data by Cussler’s model suggested that the spatial ordering of GO sheets within the main pullulan phase was perturbed because of RH fluctuations. In spite of the presence of the filler, all the formulations allowed the obtainment of final materials with haze values below 3%, the only exception being the formulation with the highest loading of GO (ϕ ≈ 0.03). The mechanisms underlying the experimental observations are discussed.

## 1. Introduction

In materials science, the advent of nanotechnology has represented a revolutionary step forward to unprecedented possibilities in the fabrication of materials and structures with outstanding functional properties. Polymer nanocomposites in particular represent one of the most successful applications of nanotechnology in different fields, including the construction, aerospace, and automotive industries, membranes, packaging materials, agri-food systems, sensors, and energy storage applications (e.g., capacitors, lithium-ion batteries, fuel, and solar cells). The advantages of nanocomposites mainly arise from the nano-length-scale entities that are used within the main polymer matrix, providing it with new performance capabilities. These nanoparticles—often named nanobuilding blocks (NBBs)—can be different in shape (round/spherical, platy, or rods/whiskers) and origin (organic or inorganic) [1,2]. If adequately manipulated, they have been proven to impart extra benefits (mechanical strength, low density, transparency, good flow, better surface properties, recyclability, etc.) as compared to conventional macrocomposites, which has been explained in terms of interface and quantum effects [3].

In some circumstances, different functional properties can be combined in a unique material for the generation of multifunctional nanocomposite polymers. Multifunctionality stems from inherent properties of the polymer matrix, of the NBBs, or a combination (often a synergy) thereof. However, multifunctionality can be suppressed by loss of structural integrity (of both the polymer phase and nanofiller) and lack of affinity at the polymer/nanofiller interface. Multifunctionality is a highly sought-after feature, especially when the high unit cost of the nanocomposite material represents a hurdle to market applications. In these circumstances, this high cost is somehow counterbalanced by the multiple functional properties offered by the material, which make any commercial exploitation more affordable. This scenario is more likely to occur in cheap markets and sectors that sell commodities. One example is the food packaging sector, where the incidence of the packaging on the total cost of the food item is very important. For most food products, the packaging cost should not exceed 10% of the total cost of the goods placed on the shelves [4,5]. Therefore, expensive and sophisticated innovations have more chances to penetrate the market if they can fulfill more than one requirement at the same time.

With special reference to carbon NBBs (e.g., graphene, carbon nanotubes, fullerenes, etc.), their multifunctionality mainly lies in the large aspect ratio, specific surface area, and electrical conductivity, besides their outstanding thermal, mechanical, and optical properties [6]. Graphene, in particular, has recently attracted tremendous interest because it allows for the attainment of nanocomposite polymer systems with improved performance, even at low loading. Their broad multifunctionality makes graphene/polymer nanocomposites appealing for a wide number of potential applications, which have recently been reviewed [7]. Although most envisaged applications rely on the electrical and mechanical properties of graphene, recent studies have emphasized the potential use of graphene as a barrier against all standard gases, owing to its impermeable atomic membrane [8]. The gas-impermeable nature of graphene has been attributed to two main factors: (i) the electron density of the aromatic ring, which is sufficient to hinder the permeation of atoms or molecules [9]; and (ii) the pore diameter of the carbon ring, which is smaller than the kinetic diameter of various gases [10,11,12]. However, it should be noted that the impermeable nature of graphene is warranted only if the large surface area of the monoatomic layer is preserved and any structural defect that may arise during the preparation or synthesis of graphene is prevented. This is not an easy task to achieve, because there are some defects due to graphene boundaries, point defects, and carbon rings with more or fewer than six carbon atoms [9].

Among others, polymer nanocomposites with oxygen barrier properties have represented one of the major fields of interest in recent years. Oxygen barrier materials are sought for several applications, such as electronic devices (e.g., light-emitting diodes (OLEDs) and liquid crystal displays (LCDs)), vacuum-insulating applications, packaging (food and pharmaceutical), and anti-oxidation layers for metals [13]. In this work, we present the development of a bionanocomposite coating with high oxygen barrier properties. More specifically, the main polymer phase was the exopolysaccharide pullulan, whose peculiar functional properties make it appealing for several applications [14]. The NBB used was graphene oxide (GO), which was selected for two main reasons: (i) to increase the interface affinity, thereby limiting unwanted phenomena such as de-bonding; and (ii) to enhance the hydrogen bonding density at the biopolymer/filler interface. The oxygen barrier performance was evaluated as a function of GO loading and different relative humidity (RH) values, and the experimental data is discussed through a modelling approach.

## 2. Results

### 2.1. Morphological Characterization of GO

**Figure 1:**
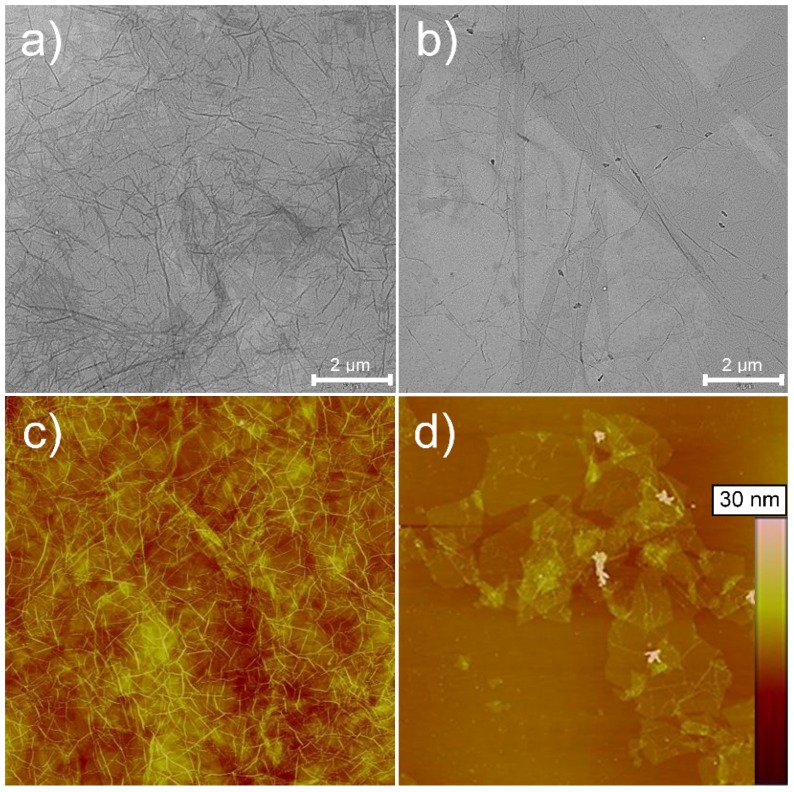
Transmission electron microscopy (TEM) images of graphene oxide (GO) at (**a**) 0.2 wt % and (**b**) 0.02 wt %. Atomic force microscopy (AFM) height images of GO: (**c**) at 0.2 wt % and 15 × 15 µm^2^; (**d**) at 0.02 wt % and 40 × 40 µm^2^. A highly wizened morphology is observed in panels (a) and (c), whereas both individual and overlapping sheets of GO are clearly visible in panels (b) and (d).

Some representative transmission electron microscopy (TEM) and atomic force microscopy (AFM) images of GO are provided in Figure 1.

Both the TEM and AFM images of GO at the highest concentration (0.2 wt %) clearly showed a “wizen-like” morphology, which was plausibly due to the mechanical stress that originated upon solvent evaporation. The same morphology was only partially visible in the diluted dispersions (0.02 wt %), where large and smooth sheets were observed, both individual and partially overlapping sheets. AFM, in particular, made it possible to calculate the average size of the GO sheets. The thickness was 1.3 ± 0.3 nm, while the width was 4.7 ± 1.6 µm, thereby suggesting an aspect ratio (α) of much larger than 3000, which is remarkably higher than natural clays, such as phyllosilicate montmorillonite [15].

### 2.2. Thickness and Oxygen Barrier Performance

Both the thickness (*l*) of bare and coated poly(ethylene terephthalate) (PET) films and oxygen transmission rate (*OTR*) data arising from the permeability tests are shown in Table 1.

As it can be seen, the actual thickness of the coatings ranged between 0.88 and 0.63 µm, thereby approaching the nominal value of 0.7 µm. Differences between theoretical and actual values can be explained in terms of different loadings of the filler, uneven deposition of the coating (due to saturation of the wire wound rod), and inherent variability of the weighing process (possibly arising from the mechanical removal of the coating from the plastic substrate). The *OTR* values of the uncoated PET decreased when RH increased. This behavior—though unusual for most plastics (especially polyolefins)—is typical for aromatic polyamides. It has been explained in terms of a decrease in both diffusivity (*D*) and solubility (*S*) of the permeant as a consequence of water molecule absorption by the film [17,18]. Overall, owing to this performance, bare PET is deemed as a medium- O_2_ barrier material. The deposition of a coating made of only pullulan allowed for a significant decrease in the permeation of oxygen across the material thickness, insomuch as the oxygen barrier performance of this polysaccharide can be compared to commonly-used oxygen barrier polymers, such as ethylene(vinyl alcohol) (EVOH), poly(vinyl alcohol) (PVOH), and polyvinylidene chloride (PDVC) at 0% relative humidity [19,20]. The *OTR* value of pullulan (1 μm thick) was estimated to be 1.73 mL·m^−2^·24 h^−1^ [21]. However, this performance tended to be less pronounced as the relative humidity increased, as with EVOH and PVOH. The same trend was observed after the addition of GO nanoparticles to the main biopolymer matrix. However, the presence of the filler dramatically improved the ultimate barrier performance—the *OTR* values measured for all the bionanocomposite-coated materials in dry conditions (0% RH) were below the sensitivity of the instrument (0.01 mL·m^−2^·24 h^−1^), with the exception of the lowest concentrated one. The best barrier performance was recorded for the formulations with the highest concentration of GO, namely those with filler volume fractions (ϕ) of 0.01341 and 0.02967. However, for all the formulations, the benefits arising from the coating deposition disappeared at 90% RH, the *OTR* value of the bare PET being restored (~100 mL·m^−2^·24 h^−1^).

### 2.3. Modelling of OTR Data

**Figure 2:**
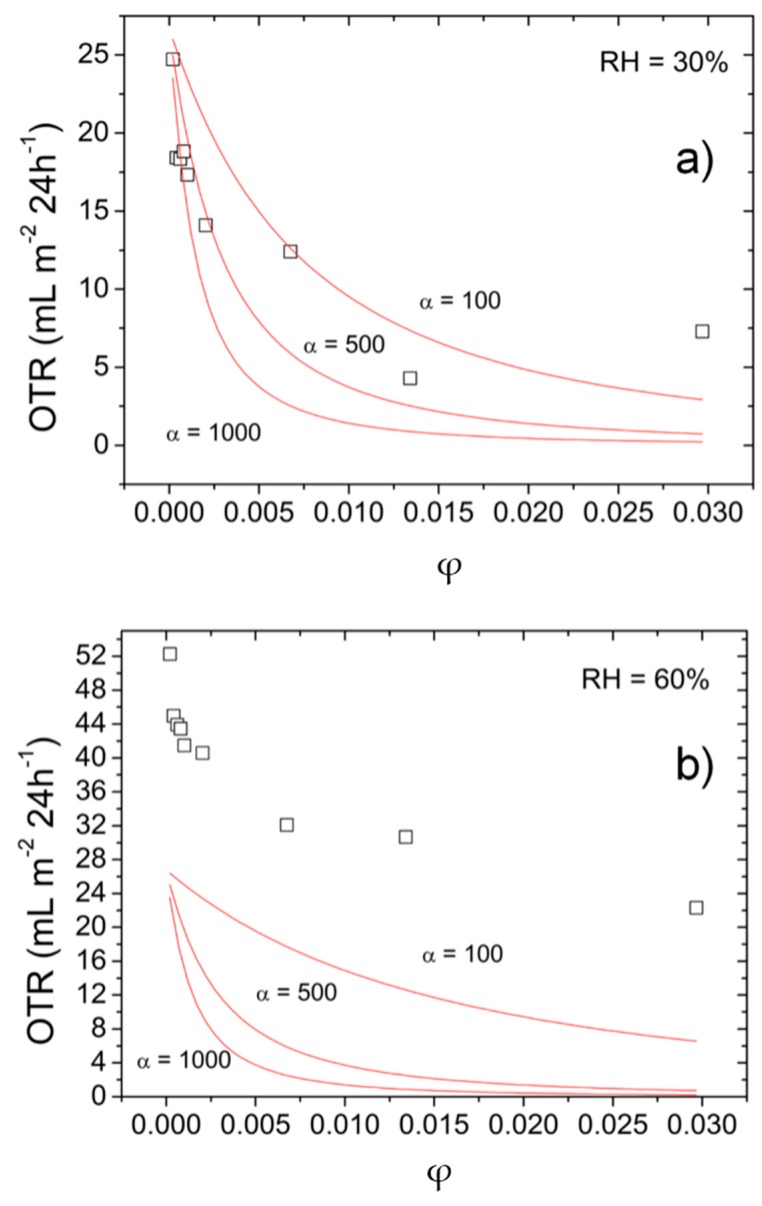
Experimental (□) and predicted (—) oxygen transmission rate (OTR) values of bionanocomposite coatings as a function of filler volume fraction (ϕ) for different aspect ratios (α) of GO platelets at (**a**) 30% RH and (**b**) 60% RH, according to Cussler’s model (Equation (1) in the text).

Modelling of the experimental permeability data is a useful approach to acquire more detailed information on the spatial organization of the filler within the main biopolymer matrix, with the goal of achieving a better interpretation of the ultimate O_2_-barrier performance of the bionanocomposite coatings as a function of perturbing factors (e.g., the relative humidity of the environment). Accordingly, in this work, we decided to use Cussler’s model for a random array of monodisperse impermeable flakes in a permeable matrix for a semi-dilute regime (i.e., αϕ >> 1) [22,23]:

(1) $$P_{0}/P\cdot \left(1-\varphi \right)={\left(1+\alpha \varphi /3\right)}^{2}$$

where *P*_0_ is the permeability parameter of the pure biopolymer coating (pullulan) (mL·m^−2^·24 h^−1^), *P* is the permeability parameter of the bionanocomposite coatings (mL·m^−2^·24 h^−1^), and α and ϕ are, respectively, the aspect ratio (the width divided by the thickness) and the volume fraction of the filler (GO) dispersed in the biopolymer matrix. Experimental and predicted data (according to Cussler’s model) for both 30% RH and 60% RH are displayed in Figure 2.

It is interesting to notice that at 30% RH, the best fitting was observed for a theoretical aspect ratio (α) of approximately 500, which is well below the experimental value of ~3000. This can be tentatively explained in terms of partial re-stacking of GO sheets in a confined environment, namely the coating thickness. At 60% RH this scenario was even more evident. The predicted data are far apart from the experimental data set, the best approaching curve being for the lowest aspect ratio (α = 200). Unequivocally, this observation suggests a change in the spatial organization of the filler, most likely given by the extensive re-aggregation of the individual GO sheets.

### 2.4. Haze

The haze values of bare PET and PET film coated with the GO/pullulan nanocomposite coatings are shown in Table 1. It is worth noting that the deposition of the coating does not impair the overall optical property. For all the coating formulations, the haze value is within the 3.0% threshold deemed necessary to warrant a suitable display of the item behind the plastic film [24]. The only exception is represented by the formulation including the highest amount of GO, for which a final haze of 3.26% was measured.

## 3. Discussion

**Figure 3:**
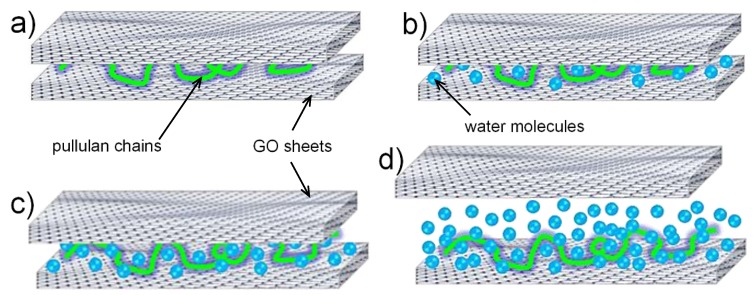
Schematic representation of the pullulan/GO nanocomposite system (**a**) in dry conditions and upon effect of relative humidity set at (**b**) 30%; (**c**) 60%; and (**d**) 90%.

The outstanding oxygen barrier performance of the GO/pullulan coatings in dry conditions is clear evidence of the impermeable nature of GO. The platy nanoparticles dispersed in the main polymer matrix acted as a physical barrier to the permeation of the oxygen molecules, which were hindered from passing through the plastic film. Aside from the physical impedance, the interaction between the organic phase and filler might also have played a role [25]. The cooperative adhesion forces at the biopolymer/filler interface mainly driven by extensive hydrogen bonding reduced any possibility for the polymer chains to experience spatial motion, resulting in a very tight and dense network, with pullulan presumably fully intercalated between the GO sheets (Figure 3a). However, the scenario changed completely because of the relative humidity, as schematically sketched in panels b–d of Figure 3. The deleterious effect of humidity on the barrier performance of nanocomposites based on hydrophilic polymers is well known and widely documented [18,26]. It is ascribed to the plasticizing effect of water molecules adsorbed by the surface and bulk of the polymer, especially in correspondence with the amorphous regions. In this study, the specific interaction between water molecules and hydrophilic filler also played a role. Due to the moisture uptake, weakening of the hydrogen bonds at intramolecular and intermolecular levels is expected. At the local level, because of the hydrophilic nature of the coating, we assume that after increasing the relative humidity to 30%, the surrounding water molecules started to interact with the bionanocomposite network, impairing the hydrogen bonding framework between pullulan and GO (Figure 3b). A further increase to 60% allowed more water molecules to interact with the bionanocomposite network, leading to an increase in the interlayer distance between overlapping GO sheets. At 90% RH, it is likely that the “plasticizing” effect of water molecules turned to a “diluting” effect. Both effects led to a dramatic increase in the free volume of the network and segmental mobility of the biopolymer chains. Eventually, the increase of RH up to 90% caused the total loss of the barrier performance of the bionanocomposite coating due to an unconstrained diffusion of the permeant across the nanocomposite polymer network. This observation is in line with our previous studies on pullulan-based coatings on PET [27,28].

**Figure 4:**
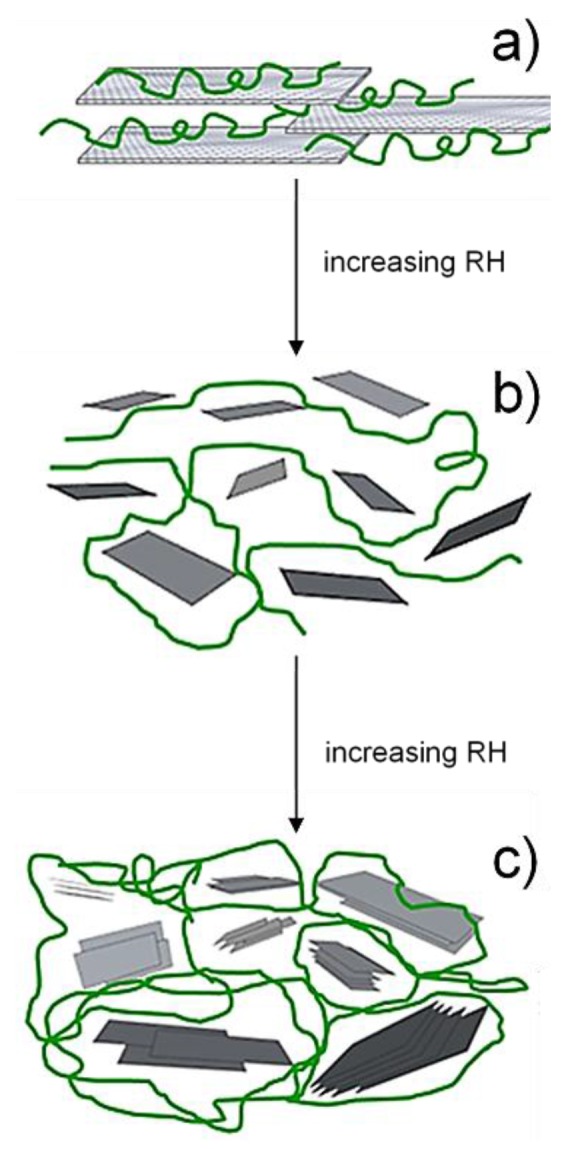
Large-scale effect of moisture uptake on the pullulan/GO nanocomposite coating: (**a**) ordered organization in dry conditions and at low RH values; (**b**) disordered organization and increased mobility due to the “dilution” effect; (**c**) discretization of the GO sheets into graphitic domains.

On a larger scale, modelling of experimental data suggests that the nanocomposite coating was organized in a relatively ordered structure under dry/low RH conditions, with the pullulan molecules intercalated within two GO sheets (Figure 4a). The “dilution” effect arising from moisture uptake yielded a disordered configuration (Figure 4b), where the mobility of both biopolymer chains and filler eventually led to a “discretization” of the GO sheets, which restored their graphitic nature (Figure 4c).

The excellent optical properties (haze) of the final nanocomposite-coated PET films recorded under dry conditions can be explained by the absence of macroscopic domains that act as scattering centers across the coating thickness. The main reason for this can be found in the high exfoliation state of GO sheets achieved by ultrasonication, besides the excellent interfacial interactions between GO and pullulan mediated by extensive hydrogen bonding. Therefore, GO monolayers encased within the main biopolymer phase allow for the achievement of striking oxygen barrier performance while maintaining almost unaltered the see-through capability of the final material. This twofold functionality—associated with ease of preparation and use of water as the only solvent throughout the process—makes pullulan/GO nanocomposite coatings a promising candidate for applications requiring barrier materials with low oxygen permeability, such as electronic and packaging (food/pharmaceutical) applications.

## 4. Materials and Methods 

### 4.1. Raw Materials and Reagents 

Pullulan (PI-20 grade, M_w_ ~200 kDa) was purchased from Hayashibara Biochemical Laboratories Inc. (Okayama, Japan). The structural characteristics of pullulan (determined by high-performance size exclusion chromatography equipped with multi-laser scattering and refractive index detectors—HPSEC-MALLS-RI) are: weight average molar mass (M_w_) = 2.094 × 10^5^ ± 0.002; polydispersity index (M_w_/M_n_) = 1.321 ± 0.02; and radius of gyration (*R_g_*) = 24.7 ± 0.002 nm [29]. As a plastic substrate, AryaPET–A410 (JBF RAK LLC, Ras Al Khaimah, United Arab Emirates), kindly provided by Metalvuoto Spa (Roncello, Italy), was used. It is a one-side corona-treated polyester film 12.0 ± 0.5 μm thick, suitable for metallizing, printing, and lamination, with good wettability and excellent machinability. Graphene oxide (2 mg/mL, dispersion in H_2_O) was purchased from Sigma-Aldrich (Milano, Italy). Milli-Q water (18.3 MΩ cm) was used as the only solvent throughout the experiments.

### 4.2. Bionanocomposite Coatings Preparation

A fixed amount of pullulan in water (10 wt %, wet basis) was prepared at 25 °C for 1 h under gentle stirring (500 rpm). In parallel, 50 mL of the as-received GO dispersion (0.2 wt %, wet basis) was ultrasonicated by an UP400S (power_max_ = 400 W; frequency = 24 kHz) ultrasonic device (Hielscher, Teltow, Germany) equipped with a cylindrical titanium sonotrode (mod. H14, tip Ø 14 mm, amplitude_max_ = 125 μm; surface intensity = 105 W·cm^−2^) under the following conditions: 0.5 cycle and 50% amplitude for 2 min. The resulting GO dispersion was used as a parental source to obtain nine batches at different GO concentrations by dilution in water (300 rpm for 15 min). The nine GO water dispersions were then mixed with a fixed amount of pullulan water solution (300 rpm × 1 h) so that the amount of GO in the final system was between 0.002 and 0.15 wt % (wet basis). Before the coating deposition, PET films were treated with a high frequency corona treatment (Arcotec, Mönsheim, Germany) with the goal of refreshing the original treatment made by the manufacturer. At this point, an aliquot (~5 mL) of each bionanocomposite water dispersion was placed on the corona treated side of rectangular (24 × 18 cm^2^) PET samples using a pipette. The deposition of the dispersion was carried out by an automatic film applicator (model 1137, Sheen Instruments, Kingston, UK) at a constant speed of 150 mm·min^‒1^, according to ASTM D823-07-Practice C, using a horizontal steel rod with an engraved pattern, which yielded final coatings of comparable nominal thickness of 0.7 µm after water evaporation. Water evaporation was achieved using a constant and perpendicular flux of mild air (25.0 ± 0.3 °C for 2 min) at a distance of 40 cm from the applicator. After drying, the concentration of GO in the nine different samples corresponded to 0.02, 0.04, 0.06, 0.08, 0.1, 0.2, 0.5, 1.0, and 1.5 wt % (dry basis). Coated films were then stored under controlled conditions (23.0 ± 0.5 °C in a desiccator) for 1 week before measurements were taken.

### 4.3. Analyses

GO particles were characterized by both transmission electron microscopy and atomic force microscopy. TEM in particular allowed for the gathering of information on the degree of exfoliation of GO sheets. To this scope, a LEO 912 AB energy-filtering transmission electron microscope (EFTEM) (Carl Zeiss, Oberkochen, Germany) operating at 80 kV was used for the analysis of graphene structures. Digital images were recorded with a ProScan 1K Slow-Scan CCD camera (Proscan, Scheuring, Germany). Samples for TEM analyses were prepared by drop-casting a few millilitres of dispersion onto Formvar-coated Cu grids (400-mesh) and letting the samples to rest for 24 h at room temperature to allow water evaporation. Atomic force microscopy experiments were carried out to quantify the size features of GO (e.g., width and thickness). The analyses were performed with a Nanoscope V Multimode (Bruker, Karlsruhe, Germany) in intermittent-contact mode after dropping 10 μL of diluted GO water dispersion (0.2 mg/mL and 0.02 mg/mL) onto a mica substrate. The images were collected with a resolution of 512 × 512 pixels with silicon tips (force constant 40 N/m, resonance frequency 300 kHz). Dimensional calculations on the acquired images were conducted with Nanoscope software (version 7.30, Bruker, Karlsruhe, Germany). The mean values reported for GO sheet dimensions were calculated over several images.

The thickness of the pullulan/GO nanocomposite coating was obtained by a gravimetric method. A 10 × 10 cm^2^ sample (coated PET) was cut and weighed (*M_1_*, grams). The coating was then mechanically removed by immersion in hot water (80 °C), and the resulting bare PET film was weighed (*M_2_*, grams). The apparent thickness (μm) of the coating was obtained according to the following equation [30]:

(2) $$l=\frac{M_{1}-M_{2}}{\rho }\times 100$$

where ρ (g·cm^−3^) is the density of the aqueous dispersion. Three replicates were analyzed for each biopolymer composition.

The oxygen barrier properties of the coated PET films were assessed using a MultiPerm permeability analyzer (Permtech Srl, Lucca, Italy) equipped with an electrochemical sensor. This instrument works according to the isostatic method, which is based on the establishment of the same total pressure on both sides of the specimen (i.e., the film). A 50 cm^2^ surface sample is placed between two semi-chambers where an inert gas (usually nitrogen) is continuously flushed. After an initial conditioning step for the sample, the test gas is then flushed. The test gas will thus permeate across the sample and be carried to the detector by the inert gas. The partial pressure of the test gas is kept constant throughout the analysis to ensure the same driving force at any given time. The oxygen transmission rate (*OTR*, mL·m^−2^·24 h^−1^) data were determined according to the ASTM standard method F1927-07, with a carrier flow (N_2_) of 10 mL·min^−1^ at 23 °C and 0, 30, 60, and 90% RH, at 1 atmosphere oxygen partial pressure difference on the two sides of the specimen. Analyses were carried out with the coated side of each sample facing the upper semi-chamber into which the humid test gas (oxygen) was flushed. For both analyses, the final values result from three replicate measurements.

The optical properties of both bare and coated PET films were assessed in terms of haze, which is a measure of the light scattered by the sample due to surface irregularities (e.g., surface roughness) [31] or bulk defects (e.g., the presence of crystalline domains) [32]. From a practical point of view, haze is linked to the “see-through” capability—the ability of a material to distinctly display an item (e.g., food products) behind it. In this work, haze was quantified according to ASTM D 1003-00 by means of a spectrophotometer coupled with an integrative sphere (150 mm diameter) able to trap the amount of the incident light (λ = 780–380 nm) diffused by the sample. Three replicates were made for each uncoated and coated film sample.

## Figures and Tables

**Table 1:** Oxygen transmission rate (*OTR*) data for uncoated poly(ethylene terephthalate) (PET) film and PET coated with pullulan/graphene oxide (GO) bionanocomposite coatings at different filler volume fractions (ϕ) for different relative humidity (RH) values.

Filler Content		*l* (µm)	*OTR* (mL•m^−2^•24 h^−1^)				Haze (%)
wt %	ϕ ^†^		0% RH	30% RH	60% RH	90% RH	
Uncoated PET	-	12.00 ± 0.08 ^c^	129.23 ± 2.6 ^a^	120.67 ± 0.9 ^a^	115.10 ± 2.76 ^a^	107.47 ± 0.74 ^a^	2.72 ± 0.08 ^abc^
PET/pullulan	-	12.75 ± 0.07 ^ab^	5.99 ± 0.02 ^b^	26.74 ± 0.29 ^b^	45.80 ± 2.65 ^c^	100.73 ± 3.23 ^c^	2.63 ± 0.22 ^abc^
0.02	0.00020	12.63 ± 0.00 ^b^	0.99 ± 0.03 ^c^	24.72 ± 2.13 ^b^	52.26 ± 0.64 ^b^	107.36 ± 0.57 ^ab^	2.49 ± 0.1 ^c^
0.04	0.00041	12.67 ± 0.05 ^b^	N.D.	18.43 ± 2.00 ^c^	44.96 ± 2.87 ^cd^	100.93 ± 4.17 ^c^	2.74 ± 0.02 ^ab^
0.06	0.00061	12.72 ± 0.00 ^ab^	N.D.	18.34 ± 0.98 ^c^	43.92 ± 0.15 ^cd^	101.69 ± 1.00 ^abc^	2.70 ± 0.13 ^abc^
0.08	0.00081	12.84 ± 0.16 ^ab^	N.D.	18.82±1.16 ^c^	43.46 ± 1.76 ^cd^	101.62 ± 3.19 ^abc^	2.57 ± 0.11 ^bc^
0.1	0.00102	12.77 ± 0.05 ^ab^	N.D.	17.32 ± 0.42 ^cd^	41.48 ± 2.77 ^cd^	101.55 ± 0.71 ^bc^	2.87 ± 0.04 ^a^
0.2	0.00203	12.78 ± 0.05 ^ab^	N.D.	14.09 ± 1.35 ^de^	40.57 ± 2.88 ^d^	99.71 ± 1.83 ^c^	2.57 ± 0.01 ^bc^
0.5	0.00675	12.88 ± 0.00 ^a^	N.D.	12.40 ± 3.56 ^e^	32.08 ± 2.41 ^e^	99.10 ± 0.10 ^c^	2.78 ± 0.05 ^ab^
1	0.01341	12.79 ± 0.10 ^ab^	N.D.	4.30 ± 1.19 ^f^	30.66 ± 2.72 ^e^	100.04 ± 4.73 ^c^	2.81 ± 0.12 ^a^
1.5	0.02967	12.80 ± 0.10 ^ab^	N.D.	7.28 ± 0.98 ^f^	22.31 ± 0.65 ^f^	103.44 ± 3.62 ^abc^	3.26 ± 0.31 ^e^

^†^ Calculated for a given GO density (ρ) = 0.981 g·cm^−3^ [16] and pullulan density (ρ) = 1 g·cm^−3^. ^abcdef^ Different superscripts within a group (i.e., within each column) denote a statistically significant difference (*p* < 0.05). Error around the mean value represents the standard deviation. N.D.: below the instrument detection limit (<0.01 mL·m^−2^·24 h^−1^).
